# Scavenger Receptors in Human Airway Epithelial Cells: Role in Response to Double-Stranded RNA

**DOI:** 10.1371/journal.pone.0041952

**Published:** 2012-08-07

**Authors:** Audrey Dieudonné, David Torres, Simon Blanchard, Solenne Taront, Pascale Jeannin, Yves Delneste, Muriel Pichavant, François Trottein, Philippe Gosset

**Affiliations:** 1 Institut Pasteur de Lille, Center for Infection and Immunity of Lille, Lille, France; 2 Université Lille Nord de France, Lille, France; 3 CNRS, UMR 8204, Lille, France; 4 Institut National de la Santé et de la Recherche Médicale, U1019, Lille, France; 5 Institut Fédératif de Recherche 142, Lille, France; 6 Service d’Hématologie-Immunologie-Cytogénétique, CH de Valenciennes, Valenciennes, France; 7 LUNAM Université, Université d’Angers, Angers, France; 8 Inserm, Unit 892, Centre de Recherche en Cancérologie Nantes-Angers, Nantes, France; 9 CNRS, Unit 6299, Angers, France; 10 Université d’Angers, CHU Angers, Laboratoire d’Immunologie et d’Allergologie, Angers, France; 11 Genomic and metabolic diseases, CNRS UMR8199, IBL, Lille, France; McMaster University, Canada

## Abstract

Scavenger receptors and Toll-like receptors (TLRs) cooperate in response to danger signals to adjust the host immune response. The TLR3 agonist double stranded (ds)RNA is an efficient activator of innate signalling in bronchial epithelial cells. In this study, we aimed at defining the role played by scavenger receptors expressed by bronchial epithelial cells in the control of the innate response to dsRNA both *in vitro* and *in vivo*. Expression of several scavenger receptor involved in pathogen recognition was first evaluated in human bronchial epithelial cells in steady-state and inflammatory conditions. Their implication in the uptake of dsRNA and the subsequent cell activation was evaluated *in vitro* by competition with ligand of scavenger receptors including maleylated ovalbumin and by RNA silencing. The capacity of maleylated ovalbumin to modulate lung inflammation induced by dsRNA was also investigated in mice. Exposure to tumor necrosis factor-α increased expression of the scavenger receptors LOX-1 and CXCL16 and the capacity to internalize maleylated ovalbumin, whereas activation by TLR ligands did not. In contrast, the expression of SR-B1 was not modulated in these conditions. Interestingly, supplementation with maleylated ovalbumin limited dsRNA uptake and inhibited subsequent activation of bronchial epithelial cells. RNA silencing of LOX-1 and SR-B1 strongly blocked the dsRNA-induced cytokine production. Finally, administration of maleylated ovalbumin in mice inhibited the dsRNA-induced infiltration and activation of inflammatory cells in bronchoalveolar spaces and lung draining lymph nodes. Together, our data characterize the function of SR-B1 and LOX-1 in bronchial epithelial cells and their implication in dsRNA-induced responses, a finding that might be relevant during respiratory viral infections.

## Introduction

Aside from its mechanical barrier function, bronchial epithelial cells (BEC) regulate inflammatory and immune responses in the lung [1]–[4]. BEC exposure to aero-contaminants such as allergens, pollutants and pathogens results in stimulation of immune responses [5]. Pattern recognition receptors, which include endocytic receptors such as scavenger receptors (SRs), and signaling receptors such as Toll-like receptors (TLRs), play a key role in this process [6], [7]. BEC express TLR1–6 and more weakly TLR9 [8]. Among TLR ligands, the strongest proinflammatory response is induced by the TLR3 agonist double stranded RNA (dsRNA), a structure produced by some respiratory viruses as replication intermediates [9]. dsRNA, as well as polyriboinosinic-polyribocytidylic (poly(I:C)), a synthetic dsRNA polymer, upregulate the expression of genes coding for the chemokines and cytokines by epithelial cells [10]. dsRNA recognition by endosomal TLR3 initiates the activation of interferon-related factor-3- and NF-κB-dependent pathways responsible for the production of interferon-stimulated genes (ISG) and inflammatory mediators, respectively [11]. Interestingly, response to TLR3 ligands and to viral infection is upregulated in inflammatory conditions (including exposure to tumor necrosis factor (TNF)-α) or during inflammatory disorders [12]–[14]. dsRNA acts as a potent adjuvant, mainly through its effect on cytokine secretion and maturation of dendritic cells [15].

Recent evidences demonstrated that TLR signaling is finely tuned by the presence of co-receptors, notably SRs. The SR family contains 8 groups classified from A to H of functionally related, but structurally heterogeneous, molecules [16], [17]. SR of the class A including SR-A have a structure of type II membrane glycoproteins which forms homotrimers. Others members of class A SR, including scavenger receptor of class A (SCARA)-2 (macrophage receptor with collagenous structure (MARCO)), 3, 4 (collectin-12 (COLEC12)) and 5, have been described as structurally related molecules although their function was not identified [16], [18]–[20]. Among class B SR, CD36 and SR-B1 are type II glycoproteins with a multiple transmembrane domain and in the extracellular domain, a loop maintained by di-sulfur links. In the class E SR, LOX-1 (lectin-like oxidised LDL receptor-1) is a type II membrane glycoprotein that includes a type C lectin domain. In the class F, the mRNA encoding SREC (scavenger receptors expressed on the endothelial cells)–1 is the source of five isoforms and the major type I membrane glycoprotein is characterized by an EGF-like domain. The class G SR, SR-PSOX (SR phosphatidylserine and oxidised lipoprotein), also called CXCL16, includes in its extracellular region, both a chemokine and a mucin domain. This extracellular region can be cleaved by metalloproteases and acts as a soluble chemokine. SRs mediate rapid internalization of bound ligands and rapidly recycle, probably through the endosomal compartment. SRs are functionally defined by their ability to recognize and to internalize modified self, such as oxidized low-density lipoproteins (LDL). Other ligands for SRs include modified (*i.e.* maleylated) proteins generated during inflammation, and pathogen associated molecular pattern, such as type D oligonucleotide sequences (ODN) and fucoidin [7], [21]. Among the receptors involved in microbe recognition, CXCL16 has been described as a coreceptor for type D ODN, a TLR9 ligand, in human plasmacytoid DC, this receptor being necessary for cell activation by this ligand [22]. CD36, LOX-1 and SREC-1 are necessary for the internalization of TLR2 ligands in antigen presenting cells [23], [24]. Although SRs are involved in the control of the immune response, their role in the lung inflammatory response is poorly defined. In BEC, the low response to TLR2 ligands, like lipoteichoic acid, is associated with defective expression of CD36 [25]. SR-A1 and MARCO ligates ligands of intracellular TLR and NOD-like receptors and allow cell activation by these compounds [26]. In addition, these receptors are involved in the defense against *Streptococcus pneumoniae*, *haemophilus influenzae* and *Neisseria meningococcus*. through their implication in bacterial endocytosis by macrophages [27]–[29]. Recently, Limmon et *al*. reported that in BEC, the scavenger receptor SR-A1 is a receptor for dsRNA [30]. In the current study, we investigated the potential role of several SRs involved in pathogen recognition during dsRNA-mediated activation of BEC. Since this was not fully described, we first analyzed the expression of these SR in BEC at steady state and in inflammatory conditions.

Our data show that BEC express various SR members and that inflammatory factors, including TNF-α, positively modulate their expression. We also report that, LOX-1 and SR-B1 play a key part in the activation of BEC by dsRNA, at least in part by controlling its uptake. Finally, SRs participate in the internalization of maleylated ovalbumin (mOVA) in BEC and *in vivo* mOVA modulates the inflammatory response triggered by dsRNA. Indeed, several studies have reported that maleylation, that introduces negative charges upon treatment with maleyc anhydride, converts macromolecules to specific ligands for SR, both in vitro and in vivo [31]–[33]. Collectively, SR/TLR3 cross-talk appears to be instrumental in the pulmonary response, a finding that might be relevant during respiratory viral infections.

## Results

### Expression of SR in Bronchial Epithelial Cells

**Figure 1:**
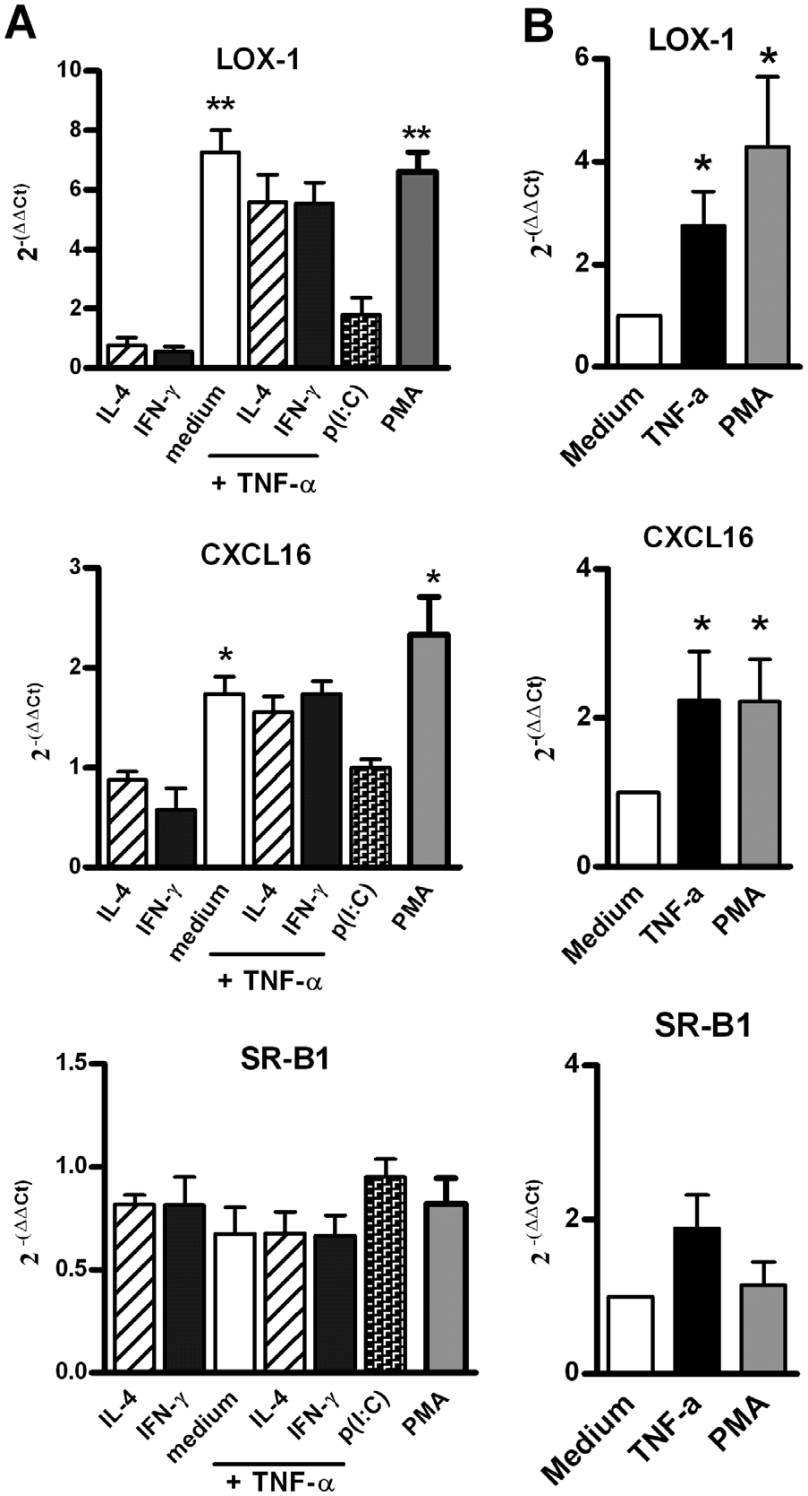
mRNA expression of SR in human BEC. (**A**) mRNA expression of LOX-1, CXCL16 and SR-B1 by stimulated 16 HBE cells. These cells were stimulated for 6 h with IL-4, IFN-γ, TNF-α, poly(I:C) and PMA. mRNA expression was analyzed by quantitative RT-PCR. Results were normalized using β-actin as endogenous control and are shown as fold changes (2^−ΔΔCt^) relative to unstimulated cells used as calibrator. Data reported the mean ± SEM from 4 independent experiments. (**B**) mRNA expression of LOX-1, CXCL16 and SR-B1 by stimulated primary cultures of BEC. Cells were stimulated for 6 h with TNF-α, and PMA. Data were expressed as mean ± SEM of 2^ -ΔΔCt^ from 4 independent experiments. *: p<0.05, **: p<0.01 versus control cells.

The expression of major SR involved in the recognition of pathogens was first analysed by quantitative RT-PCR in immortalized (16 HBE) and primary (HBEC) bronchial epithelial cells under resting conditions and in different conditions of stimulation reproducing inflammatory environments. Under resting conditions, the level of transcripts specific for SR-B1 and CXCL16,was high and it was found to be intermediate for LOX-1 and SREC-1 (Table 1). Among class A SR, SR-A1 and SCARA5 transcripts were not detected in BEC unlike in peripheral blood mononuclear cells (Table 1 and data not shown). COLEC12 and MARCO were expressed at a low level close to the one observed for SREC-1, whereas the level of expression for SCARA3 (variant 1) was similar to that of SR-B1 and was higher in BEC, as compared with mononuclear cells (Table 1). We next evaluated the effect of cytokines, TLR ligands and of the protein kinase C activator PMA on SR expression. TNF-α and PMA promoted an increased expression of LOX-1 and, to a lesser extent, of CXCL16 on 16 HBE cells and HBEC with a peak at 3–6 hrs post-stimulation (Fig. 1 and data not shown). In contrast, TNF-α and PMA had no effect on COLEC12, MARCO, SCARA3, SCARA5, SR-B1, SRA1 and SREC-1 transcript expression (Fig. 1 and not shown). Neither interleukin (IL)-4, interferon (IFN)-γ (alone and with TNF-α) nor poly(I:C), Pam_3_CSK_4_, LPS, CpG ODN affected transcription of SR genes (Fig. 1A and not shown).

**Table 1:** mRNA expression for SR in immortalized cells (16 HBE), primary culture of bronchial epithelial cells (HBEC) and peripheral blood mononuclear cells (PBMC) under resting condition.

	16 HBE	HBEC	PBMC
**SR-A1**	>25	>25	11.3±1.4
**SR-B1**	5.3±0.5	6.2±0.9	9.3±0.8
**CXCL16**	7.5±0.7	5.2±0.9	5.8±0.9
**LOX-1**	12.8±1.3	10.9±1.7	7.2±0.9
**SREC-1**	15.1±2	16.1±1.5	14.4±1.4
**MARCO**	23.1±2.1	13.4±0.85	12.6±2.5
**COLEC12**	19.3±1.4	18.1±2.2	12.5±0.3
**SCARA3**	8.5±1.1	5.4±1.2	19.4±1.9
**SCARA5**	24.1±1.2	18.7±1.5	22.4±0.8

The results obtained by QRT-PCR were expressed as ΔCT between the Ct obtained for the SR and β-Actin. The higher is the ΔCT, the lower mRNA expression level is (n = 4; mean ± SEM).

**Figure 2:**
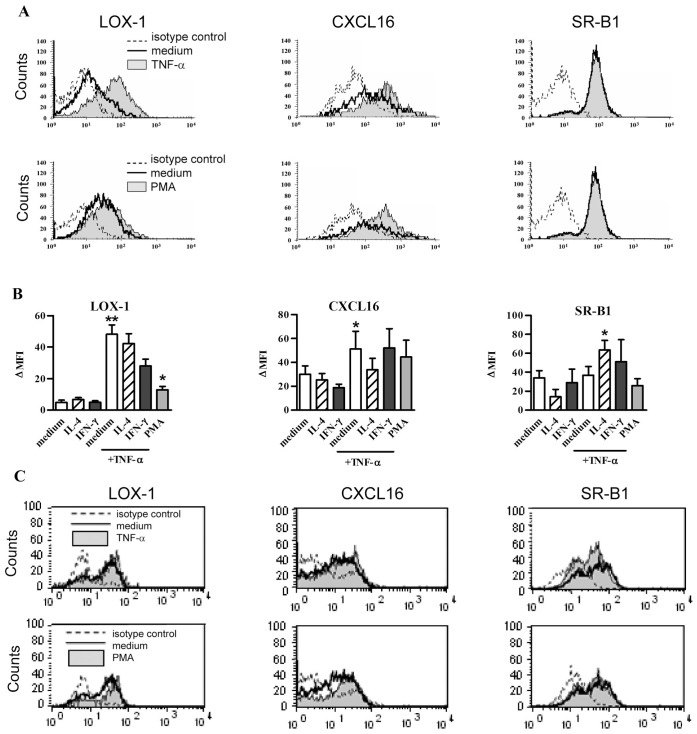
LOX-1, CXCL16 and SR-B1 surface expression in BEC. (**A**) Analysis of SR expression by flow cytometry in 16 HBE cells. These cells were stimulated for 24 h with TNF-α (upper panel) or PMA (lower panel) (filled line, upper and lower histogram, respectively) as compared with cells in medium (bold line). Isotype control is represented with dotted line. Histograms of one representative experiment out of 7 are presented. (**B**) Modulation by cytokines and PMA of LOX-1, CXCL16 and SR-B1 expression in 16 HBE cells. The mean ± SEM of ΔMFI for 7 independent experiments are reported. *: p<0.05, **: p<0.01 versus control cells. (**C**) Modulation of LOX-1, CXCL16 and SR-B1 expression in HBEC stimulated for 24 h with TNF-α (upper panel) or PMA (lower panel). Histograms of flow cytometry are reported for HBEC. The filled line showed the stimulated cells as compared with cells in medium (bold line) and to the isotype control (dotted line). This is a representative experiment out of 3.

As assessed by flow cytometry, the expression in steady state conditions of SR-B1 was high, CXCL16 intermediate, and LOX-1 very low on 16 HBE cells (Fig. 2). On the other hand, MARCO, SR-A1 and SREC-1 were undetectable on 16 HBE cells and HBEC whereas these antibodies recognized CHO cells transfected with the corresponding genes (Figure S1). The expression of LOX-1 and CXCL16 was increased after treatment with TNF-α and PMA (Fig. 2A–B). In contrast, IL-4 and IFN-γ alone, or combined with TNF-α did not modulate LOX-1 and CXCL16, as well as SR-B1 expression (not shown). In agreement with Fig. 1, TLR agonists, including p(I:C), did not affect SR protein expression (data not shown). On HBEC, the expression of LOX-1, CXCL16 and SR-B1 was not significantly modulated by TNF-α or PMA (Fig. 2C). Collectively, our data demonstrated that SR members are differentially expressed by BEC and that the synthesis of LOX-1 and CXCL16 can be modulated by TNF-α or PMA.

### BEC Uptake SR Ligands

**Figure 3:**
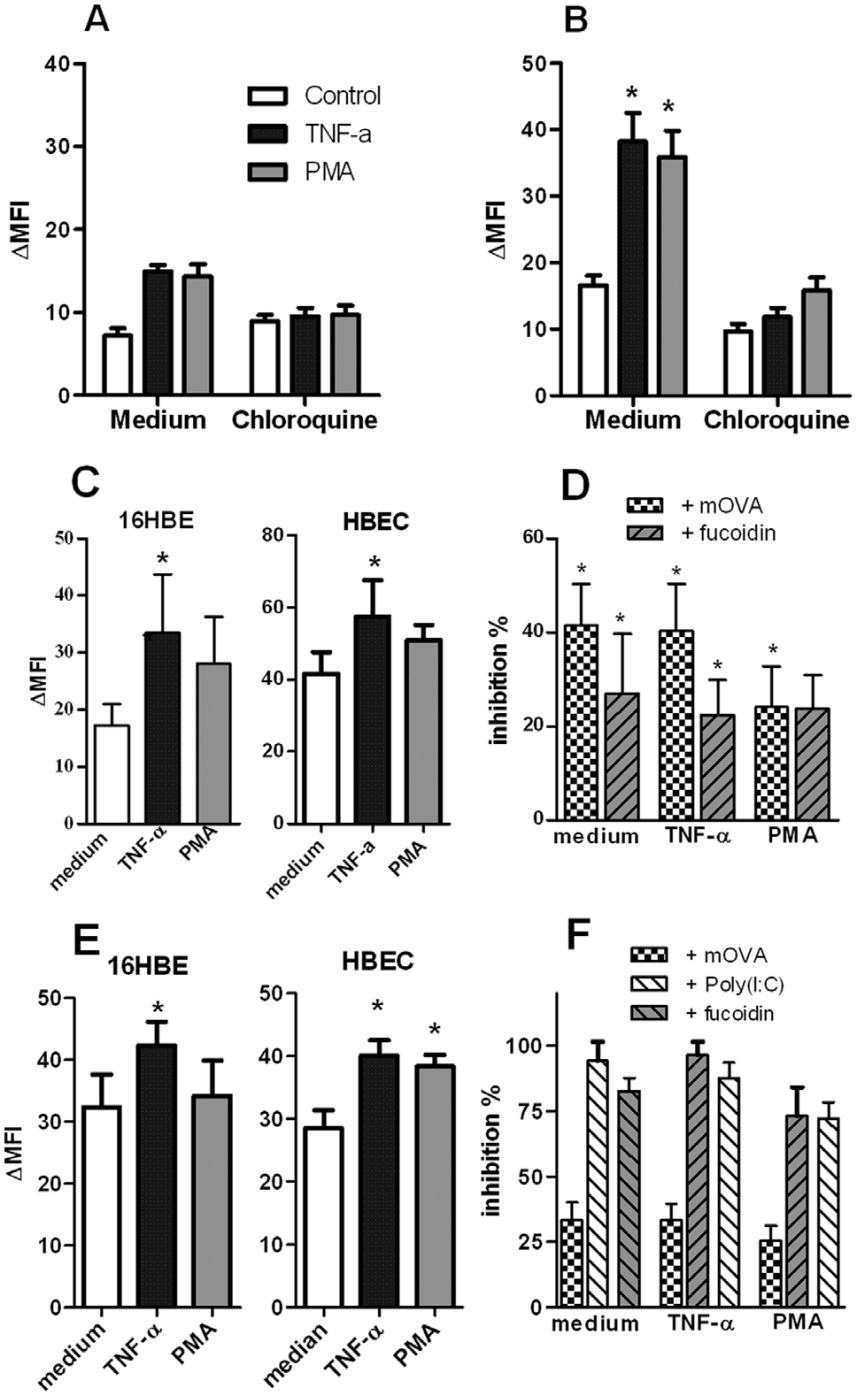
Endocytosis of SR ligands and dsRNA by BEC. (**A–B**) Uptake of AlexaFluor®488-labelled Ac-LDL by 16 HBE cells. 16 HBE cells were stimulated with TNF-α (20 ng/mL) or PMA (10 ng/mL), in the absence or presence of chloroquine (100 µM), and then, were exposed for 30 minutes (A) or 1 hour (B) with labelled-Ac-LDL (10 µg/mL). The binding of the ligand was assessed by flow cytometry (n = 3). (**C**) mOVA binding is measured in 16 HBE cells and HBEC activated for 24 h with TNF-α and PMA. Cells were incubated for 1 h with FITC-conjugated mOVA. MFI was determined by flow cytometry. (**D**) Inhibition of mOVA uptake by unlabelled SR ligands. An excess of unlabelled mOVA and fucoidin was added 10 min before addition of FITC-labelled-mOVA. Data were expressed as the mean ± SEM from 5 to 13 independent experiments. (**E**) Modulation by TNF-α and PMA of the biotinylated poly(I:C) endocytosis in 16 HBE cells (left panel) and HBEC (right panel). Data are expressed as the mean ± SEM of ΔMFI (n = 4 to 5 experiments). (**F**) Inhibition of poly(I:C) binding by mOVA, fucoidin and unlabelled poly(I:C) on 16 HBE cells activated or not with TNF-α and PMA. Mean ± SEM of the percentages of inhibition were reported (n = 3). *: p<0.05, **: p<0.01 versus control cells.

We next evaluated the activity of SR in BEC by quantifying the uptake of SR ligands. 16 HBE cells were labelled 30 and more markedly, 60 min post incubation with FITC-labelled Ac-LDL (Fig. 3A–B). TNF-α or PMA treatment increased by 2-fold the level of binding. Unlabelled Ac-LDL and fucoidin, another SR ligand, inhibited the binding of labelled Ac-LDL by 81 and 72%, respectively. Chloroquine, an endosomal inhibitor, markedly decreased FITC labelling (Fig. 3A–B). In the same line, FITC-labelled mOVA, an SR ligand mimicking denaturated protein, was internalized by 16-HBE cells (Figure 3C, left panel) and HBEC (Figure 3C, right panel) after 60 min. Activation with PMA and TNF-α upregulated the binding of mOVA in both cell types as illustrated in HBEC (Figure S2). Unlabelled mOVA and fucoidin significantly inhibited the endocytosis of mOVA by 30 to 40% (Fig. 3D). In contrast, addition of native ovalbumin did not affect the binding of FITC-labelled mOVA (data not shown).

We next investigated the capacity of SRs to bind dsRNA. Unstimulated cells constitutively bind poly(I:C) and pre-treatment with TNF-α, but not PMA, significantly increased it (Fig. 3E). Addition of fucoidin and unlabelled poly(I:C) markedly inhibited the uptake of biotinylated poly(I:C) on BEC whereas mOVA had a lower effect (Fig. 3F). Collectively, BEC can take up SR ligands, including Ac-LDL and mOVA, as well as dsRNA, a phenomenon amplified by TNF-α and to a lesser extent by PMA.

### Maleylated Ovalbumin Reduces the Poly(I:C)-induced Signaling and Affects Cytokine Production by BEC

**Figure 4:**
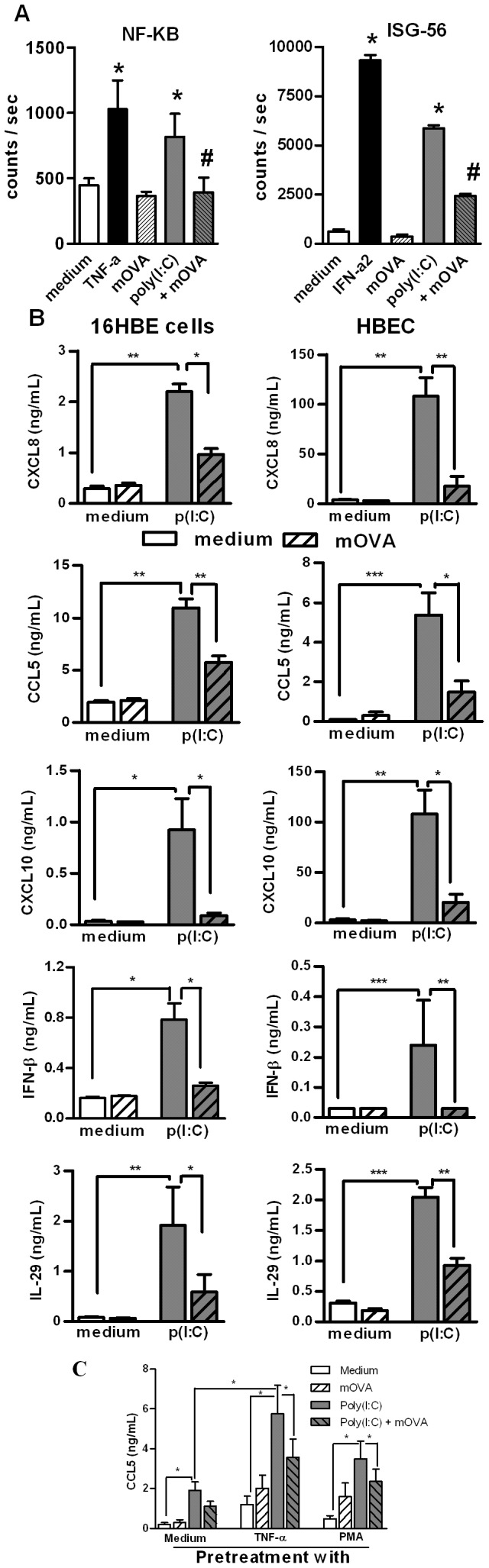
Modulation by SR ligand of poly(I:C)-induced activation in BEC. (**A**) Inhibition by mOVA of dsRNA-induced NF-κB-dependent and ISG-56-dependent luciferase production in BEAS-2B cells transfected with the appropriate plasmid. The values obtained with medium and mOVA alone, with positive control (TNF-α or IFN-α2) were also reported. Results were expressed as mean ± SEM of counts per second (n = 3). *: p<0.05 versus cells in medium alone; ≠: p<0.05 versus cells with poly(I:C). (B) Addition of mOVA modulated dsRNA-induced cytokine secretion in 16 HBE cells and HBEC. Secretion of CXCL8, CCL5, CXCL10, IFN-β and IL-29 was measured in supernatants of BEC in medium alone or stimulated with p(I:C) (mean ± SEM from 6 to 7 independent experiments). * p<0.05, ** : p<0.01; ***: p<0.001. (**C**) Pretreatment of 16 HBE cells with TNF-α and PMA increased the production of CCL5 in response to poly(I:C). Addition of mOVA inhibited the dsRNA-induced production in TNF-α and PMA-pretreated cells. * p<0.05, n = 4.

In order to determine the role of SRs in dsRNA-induced signalling pathways, we analysed the activity of TLR3-dependent downstream regulatory elements NF-κB and ISG-56. To this end, we used the bronchial epithelial cell line BEAS-2B cells that can be efficiently transfected with reporter plasmids carrying a luciferase gene reporter regulated either by NF-κB- or ISG-56-responsive elements. BEAS-2B cells expressed SR-B1, LOX-1 and CXCL16; TNF-α increasing the expression of the last two receptors (data not shown). As shown in Fig. 4A, TNF-α and IFN-γ, used as positive controls, respectively induced NF-κB- and ISG-dependent luciferase activities. Poly(I:C) increased both NF-κB- and ISG-dependent luciferase activities, compared to untreated cells. Interestingly, pre-treatment of BEC with mOVA reduced the poly(I:C)-induced luciferase activity of the two reporter genes by 45 and 43%, respectively. These results showed that addition of a SR ligand affects the dsRNA-induced mobilization of the two signalling pathways NF-κB and IRF3.

The interference of SR in dsRNA-induced cytokine production was then evaluated in quiescent and pre-activated 16 HBE cells and HBEC. Addition of poly(I:C), but not mOVA, alone triggered the production of CXCL8, CCL5, CXCL10, IFN-β and IL-29 by both 16 HBE cells and HBEC (Fig. 4B). Interestingly, the addition of mOVA significantly inhibited the poly(I:C)-induced cytokine secretion (90-50% inhibition) (Fig. 4B). Fucoidin also decreased cytokine production induced by poly(I:C) (data not shown). Pre-activation of 16 HBE cells with TNF-α and PMA enhanced cytokine production in response to poly(I:C) as illustrated for CCL5 (Fig. 4C). This effect was inhibited by addition of mOVA (p<0.05). Collectively, the SR ligand mOVA competes with dsRNA in order to inhibit the TLR3-mediated activation of BEC.

### LOX-1 and SR-B1 are Involved in the Activation of BEC by Poly(I:C)

**Figure 5:**
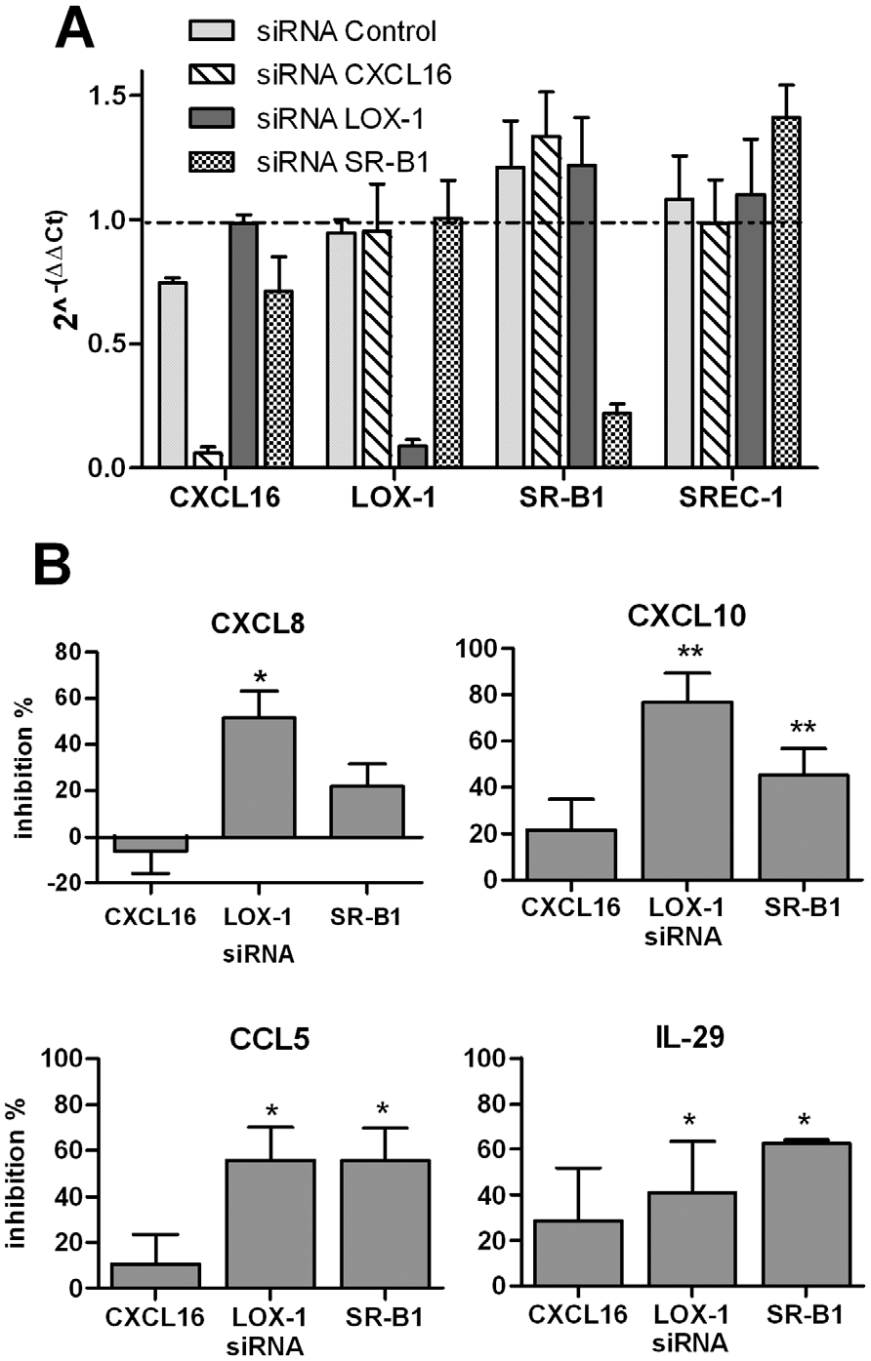
Impact of SR gene silencing on poly(I:C)-induced cytokine secretion by BEAS-2B cells. BEAS-2B cells were treated with siRNA targeting CXCL16, LOX-1 and SR-B1 or the control siRNA (25 nM) and then activated with poly(I:C). (**A**) Inhibitory activity of RNA silencing on CXCL16, LOX-1, SR-B1 and SREC-1 mRNA expression was controlled by QRT-PCR. The reference corresponds to untreated cells (value  = 1). (**B**) RNA silencing of these SR inhibits the secretion of CXCL8, CXCL10, CCL5 and IL-29 induced by p(I:C). Data were expressed as the % of inhibition as compared with cells treated with the control siRNA (mean ± SEM) (n = 5). * p<0.05, n = 4.

The role of SRs in dsRNA-induced signalling pathways was next studied using a RNAi silencing approach. CXCL16, LOX-1 and SR-B1 small interfering RNA (siRNA), but not the control siRNA, specifically inhibited the mRNA expression of the respective SR (75–90% inhibition), but not of the unrelated SREC-1 (Fig. 5A). Of interest, inhibition of LOX-1, and to a lesser extent SR-B1, expression significantly decreased the poly(I:C)-induced production of CXCL8, CXCL10, CCL5 and IL-29 (Fig. 5B). The production of IFN-γ was also significantly reduced by LOX-1 and SR-B1 silencing (39 and 62% inhibition, respectively). In contrast, treatment with CXCL16 siRNA had no significant effect. These results show that both LOX-1 and SR-B1 play a major role in the control of dsRNA-induced BEC activation.

### Intranasal Administration of mOVA Affects the Pulmonary Response to Poly(I:C)

**Figure 6:**
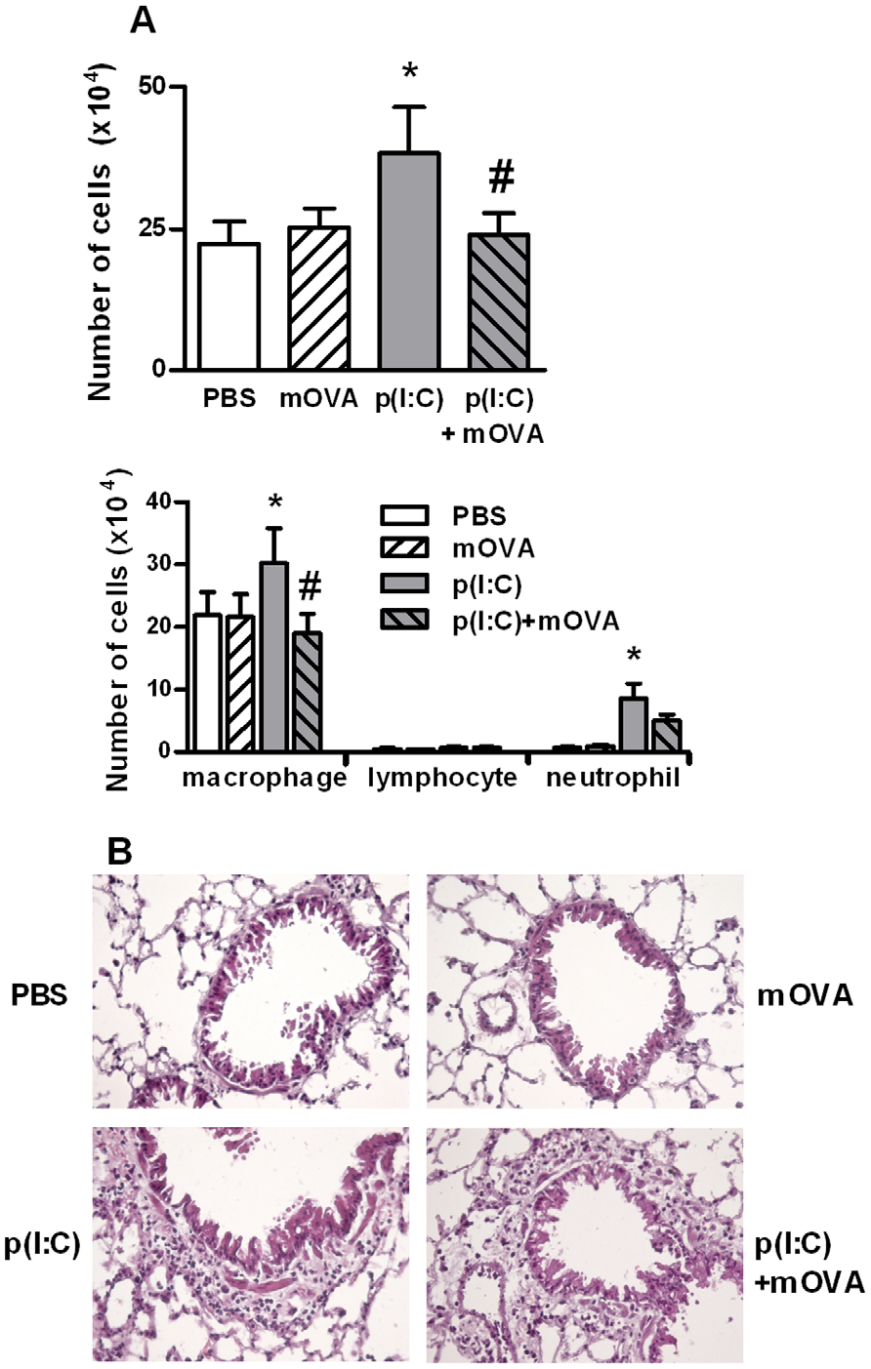
Administration of mOVA affects the acute effect of poly(I:C) on cell recruitment in BAL. Mice received one intranasal injection of PBS, mOVA (100 µg), poly(I:C) (20 µg) or mOVA + poly(I:C). BAL fluids and lungs were collected 16 h later. (**A**) Total and differential cell counts in the BAL were assessed by MGG coloration. Data are expressed as the mean ± SEM from 3 independent experiments including four mice per group. *: p<0.05 versus cells in medium alone; ≠: p<0.05 versus cells with poly(I:C). (**B**) Representative hematoxylin and eosin staining of lung sections in the four groups of mice. Magnification: ×300.

In order to determine the *in vivo* relevance of our data, we investigated the role of SRs in the early pulmonary response to poly(I:C). Administration of poly(I:C), but not mOVA, increased the total number of cells in BALs (Fig. 6A) and in peribronchial area of the lung tissue (Fig. 6B), particularly macrophages and neutrophils. Remarkably, co-administration of mOVA and poly(I:C) strongly decreased the recruitment of macrophages and neutrophils into the BALs (Fig. 6A). No recruitment of T lymphocytes and dendritic cell was detected using this protocol. In BAL fluids, the enhanced levels of CXCL10 (a chemokine active on mononuclear cell) and KC (a chemokine recruiting neutrophils) triggered by poly(I:C) were inhibited by 35 and 42% respectively by mOVA (data not shown). In contrast, treatment with mOVA had no effect on poly(I:C)-induced peribronchial cell recruitment (as illustrated in Figure 6B) and chemokine production (data not shown).

**Figure 7:**
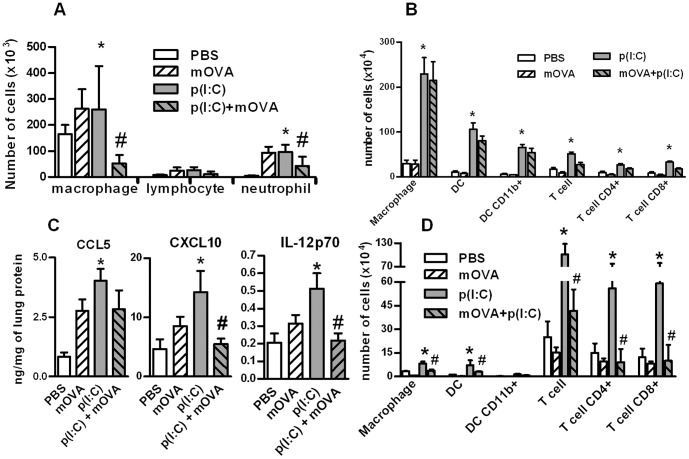
Repeated administration of mOVA affects the late response to poly(IC) in the lung. Mice received three intranasal injections of PBS, mOVA (100 µg), poly(I:C) (20 µg), mOVA + poly(I:C). BAL, lungs and lymph nodes were collected 24 h after the last injection. (**A**) Absolute numbers of cells in the BAL were reported. (**B**) Numbers of macrophages, dendritic cell (DC) (total number and CD11b^+^) and T lymphocytes (total number, CD4^+^ and CD8^+^ cells) in the lung are shown. (**C**) Levels of CCL5, CXCL10 and IL-12p70 were measured by ELISA in protein extracts from the lungs. Results were expressed as the ratio of the total concentration of protein. (**D**) Numbers of macrophages, dendritic cell (DC) (total number and CD11b^+^) and T lymphocytes (total number, CD4^+^ and CD8^+^ cells) in mediastinal lymph nodes were evaluated by flow cytometry. Data are expressed as the mean ± SEM from 3 independent experiments including 4 mice per group. *: p<0.05 versus cells in medium alone; #: p<0.05 versus cells with poly(I:C).

We also evaluated lung inflammation in a more chronic situation where poly(I:C) and/or mOVA were inoculated in a daily manner for three consecutive days. Indeed, mOVA strongly decreased the poly(I:C)-induced recruitment of macrophages and neutrophils in the BALs (Fig. 7A). mOVA also decreased CXCL10, IL-12p70, and to a lesser extent CCL5, production in the lung tissue (Fig. 7C). Repeated administration of dsRNA markedly increased the recruitment of dendritic cells (the total number and the CD11b^+^ subset), macrophages (CD11c^+^ and F4/80^+^) and CD4^+^ and CD8^+^ T cells and the expression of activation markers in lung tissue and in mediastinal lymph nodes (Fig. 7B,D and Figure S3). In draining lymph nodes but not in the lungs, mOVA strongly decreased the poly(I:C)-induced recruitment and activation of dendritic cells, macrophages and T lymphocytes. Collectively, these results showed that the SR ligand mOVA competes with poly(I:C) *in vivo* to decrease cell mobilization and activation in the lungs.

## Discussion

In this study, we evaluated the expression of SRs in BEC under steady-state and inflammatory conditions. We demonstrated that some SR members are expressed on resting BEC and that TNF-α enhances the expression of LOX-1 and CXCL16 and their function. Moreover, we showed that SRs, including LOX-1 and SR-B1, participate in BEC activation triggered by the TLR3 ligand dsRNA *in vitro*. Finally, we reported that exogenous mOVA, an SR ligand, competes to limit the dsRNA uptake by BEC and the host pulmonary response induced by dsRNA.

In steady-state conditions, BEC differentially express SR members. SRB1 and CXCL16 were strongly expressed by BEC whilst LOX-1 and CD36 were present at a lower level (both at the transcript and protein levels). Among the class A receptors, transcripts for SR-A, MARCO, COLEC12 and SCARA5 were either undetectable (ΔCt >25) or expressed at low levels (ΔCt ≈ 20). Interestingly, transcript 1 of SCARA3 is expressed at high level in BEC as compared to mononuclear cells. At the protein level, SR-A1, MARCO and SREC-1 were not detectable by flow cytometry on BEC, unlike macrophages and dendritic cells [34]. Of note, whilst transcript for LOX-1 was detected, protein expression was low in bronchial cell lines. At baseline, a higher level of these SRs was detected in HBEC as compared with 16 HBE cells. Indeed, *in vivo* constitutive activation of HBEC can be suspected due to the characteristics of the donors (smokers and/or patients with lung cancer). The influence of inflammatory mediators on SR expression was next assessed. Previous reports have demonstrated that CXCL16 and LOX-1 expression is inducible by inflammatory signals, including IFN-γ and TNF-α in macrophages, vascular cells and/or smooth muscle cells [35], [36]. In the current study, TNF- α also increased CXCL16 and LOX-1 (but not the other tested SR) expression in BEC but no synergistic or additive effects of IFN-γ was observed. Interestingly, none of class A SRs are modulated by TNF-α and PMA in BEC. Other immunoregulatory cytokines, including IL-4, did not affect the SR expression in BEC whereas IL-10 blocks the effect of TNF-α on endothelial cells [37]. PMA reproduced the major effects of TNF-α probably through the mobilization of common signaling pathways such as NF-κB. Whereas TNF-α as well as IL-4 and IFN-γ did not change SR-B1 mRNA expression, it has been reported that PPAR-γ plus RXR ligands amplified its expression demonstrating the role of other signaling pathways in the control of SR-B1 gene expression [38]. Of note, in primary cultures of HBEC, the TNF-α-induced increase of LOX-1 and CXCL16 mRNA expression was not observed at the protein level. This might be related, in HBEC, to different posttranscriptional modulation of mRNA or to an enhanced recycling of SRs through the endosomal compartment as compared with 16 HBE cells [16].

SRs have been identified for their capacity to recognize and to capture modified LDL. Indeed, SR expression on BEC was associated with an internalization of acetylated LDL and mOVA, confirming the functionality of these receptors. Chloroquine inhibited acetylated LDL internalization, indicating an endocytosis-dependent pathway. Fucoidin, which shares a binding inhibition pattern consistent with class A, B or C activity [39], strongly inhibits the uptake of mOVA and acetylated LDL to BEC. The analysis of the receptor(s) involved in mOVA uptake indicates that CHO cells transfected with the gene encoding for CXCL16, LOX-1, SREC-1 and SR-B1 strongly bind FITC-conjugated mOVA (not shown). Since mOVA also inhibited the binding of ligand for class A receptors, such as acetylated-LDL (not shown), we cannot exclude the implication of class A receptors (particularly SCARA3) in the binding of mOVA. These data demonstrate that different receptors belonging to several classes of SRs are involved in mOVA uptake by BEC. Upregulation of LOX-1 and CXCL16 expression on 16 HBE cells by PMA or TNF-α was also associated with a higher endocytosis of mOVA. This effect was also observed in HBEC, although cell activation did not upregulate the cell-surface expression of these SRs. This strengthens the hypothesis that the recycling of SRs towards the endosomal compartment, but not the cell membrane expression of these receptors, might control the kinetic of SR ligand uptake by HBEC. Collectively, BEC express SR members and uptake SR ligands including mOVA, a phenomenon that might participate in the clearance of modified proteins in the lungs and that may be accelerated during inflammatory conditions.

SRs have been described to act as co-receptors for TLR2, TLR4 and TLR9 [22], [23], [40]. In this study, we evaluated the potential implication of SR in BEC activation in response to the TLR3 ligand poly(I:C). We and others have demonstrated in BEC that chemokine production induced by dsRNA is TLR3-dependent, whereas the production of type I and III IFN is under the control of both RNA helicases and TLR3 [41]–[43]. We first observed that addition of SR ligands (mOVA and fucoidin) decreased the endocytosis of poly(I:C) and also inhibited the cytokine production induced by poly(I:C) in BEC. We have also controlled by ELISA that mOVA was not able to bind to immobilized dsRNA (data not shown) supporting the idea that mOVA does not directly interact with dsRNA. These data suggest that exogenous SR ligands compete with TLR3 agonists to control the activation of BEC. SRs may direct dsRNA into endosomes containing TLR3 and/or in the cytoplasm containing RNA helicases. The use of RNA silencing showed that LOX-1 and SR-B1 mainly controls dsRNA-induced BEC activation. Surprisingly, CXCL16 silencing did not significantly decrease cytokine production induced by dsRNA, in contrast to CpG ODN, a TLR9 ligand [22]. Treatment with mOVA inhibited the dsRNA-dependent activation of the two signaling pathways IRF3 and NF-κB as well as the subsequent production of NF-κB-dependent (CXCL8, CCL5) [44] and IRF3-dependent (CXCL10 and IFN type I and III) cytokines [45]. According to these data, the secretion of these cytokines was inhibited by LOX-1 and SR-B1 RNA silencing. The mild inhibitory effect of mOVA on poly(I:C) uptake also supports the hypothesis that SRs may facilitate TLR3 signaling as reported for TLR2 [23]. Several studies reported that class A SR including SR-A1, MARCO and SCARA3-5 are implicated in the response to dsRNA in different cell types, including BEC [26], [30], [46]. Although we were not able to detect SR-A1 and MARCO protein expression by flow cytometry, we cannot exclude the implication of these receptors in the response to dsRNA. Together, our data suggest that LOX-1 and SR-B1 particpates in the cellular uptake of poly(I:C) by BEC, a process that amplifies the dsRNA-induced inflammatory response. In addition, the TNF-α-induced increase of the cytokine secretion in response to poly(I:C) may be related to the enhanced expression of SR such as LOX-1 and to the increased uptake of this PAMP.

We next analyzed the effects of mOVA, used here as a competitor, on the poly(I:C)-induced pulmonary responses. In the two models used, poly(I:C) induced an early influx of neutrophils and macrophages within the alveolar spaces. Repeated administration of dsRNA also led to the recruitment of dendritic cells and lymphocytes and to their subsequent activation. In these models, treatment with mOVA strongly decreased the dsRNA-induced mobilization of macrophages and neutrophils in the BALs. Similarly, mOVA decreased the migration of dendritic cell and lymphocytes in the mediastinal draining lymph nodes. Of note, administration of mOVA did not decrease cell recruitment and activation in the lung tissue suggesting that mOVA preferentially affects the cell relocation in the lung. This may be related to an altered production of chemokines in the alveolar spaces and to an altered maturation of pulmonary dendritic cell limiting their emigration towards the LN. In this context, mOVA treatment diminished the poly(I:C)-induced lung production of bioactive IL-12, a pro-Th1 associated cytokine mainly produced by dendritic cells. Collectively, our data indicate that mOVA binding to SR partially blocked the lung inflammatory response to poly(I:C). Since the implication of SR-A and MARCO has been previously demonstrated in the lung inflammatory response to dsRNA [26], [30], [46], we can suspect that these receptors, expressed at high levels in mononuclear phagocytes, are involved in the response of macrophages to dsRNA, whereas SR-B1 and LOX-1 are prominent in the response of airway epithelium. Altogether, SRs might serve as co-receptors for dsRNA to regulate the recruitment of inflammatory cells into the lungs and the activation of T cells and dendritic cells in draining lymph nodes.

To conclude, both *in vitro* and *in vivo*, SRs act as carriers, facilitating dsRNA entry and delivery to the dsRNA-sensing receptors in BEC. Whilst SRs including SR-B1 and LOX-1 might serve as co-receptors for TLR3, the presence of specific SR ligands in the surrounding milieu might inhibit the response to TLR3 ligand. These data also suggest that these receptors recognize danger signals associated with lytic virus infections. Indeed, respiratory viruses produced dsRNA and these infections are associated with the production of SR ligands released by dead cells probably including modified proteins during the death process (oxydation, sugar modification). Whether or not generation of endogenous SR ligands inhibit immune responses following infection with dsRNA-producing respiratory viruses is still unknown and worth of future studies. In the same line, members of the SR family might represent novel targets for therapeutic or preventive interventions that aim to control virus-associated immunopathologies.

## Materials and Methods

### Cell Cultures and Activation

16 HBE and BEAS-2B cells were obtained from the ATCC (Manassas, VA). 16 HBE cells were grown in DMEM supplemented with 10% heat-inactivated FCS, L-glutamine and antibiotics (Life Technologies, Courtaboeuf, France). BEAS-2B cells were maintained in airway epithelial cell growth medium (Promocell, Heidelberg, Germany). Cell lines were cultivated on culture plates coated with human collagen G matrix (Biochrom, Berlin, Germany). Human bronchial epithelial cells (HBEC) were prepared from bronchial wall explants dissected in healthy area of lung tissues obtained from patients undergoing partial or complete lung resection (n  = 6). Lung resection was performed for lung cancer. All the patients were former smokers or smokers who stopped smoking before surgery. Tissue samples were initially collected for the study PHRC N°2002-1916, approved by the CPP (comité de protection des personnes) and signed informed consent has been obtained for each subject. HBEC were starved overnight with basal medium and then activated by different stimuli. For the analysis of SR expression, BEC were exposed after cell starvation to TLR ligands including poly(I:C) (10 µg/ml), Pam_3_CSK_4_, LPS and CpG (1 µg/ml) and to cytokines including IL-4, TNF-α and IFN-γ (10 ng/ml). In order to determine the implication of SRs, cells were exposed to mOVA (50 µg/ml) [24] before activation by dsRNA (1 or 10 µg/ml). Details are provided in File S1.

### Reverse Transcriptase-Polymerase Chain Reaction (RT-PCR) Analysis

Quantitative RT-PCR was performed to quantify the housekeeping gene β-actin, LOX-1, SR-A1, SR-B1, SREC-1 and CXCL16 mRNA. Forward and reverse primers were designed as described in Table 2. Results were expressed as mean ± SEM of the relative gene expression calculated for each experiment in folds (2^−ΔΔCt^) using β-actin as a gene reference and compared to unstimulated cells used as calibrator.

**Table 2:** Sequences of primers for qRT-PCR.

Target	Primer	Sequence
**β-Actin**	Forward	5′-CTG GAA CGG TGA AGG TGA CA
	Reverse	5′-AAG GGA CTT CCT GTA ACA ATG CA
**LOX-1**	Forward	5′-TTG TCC GCA AGA CTG GAT CTG
	Reverse	5′-TGG CAT CCA AAG ACA AGC ACT
**SR-A1**	Forward	5′-TTC AAA GCT GCA CTG ATT GCC
	Reverse	5′-TTC TTC GTT TCC CAC TTC AGG A
**SR-B1**	Forward	5′-TTT GAA GGC ATC CCC ACC TA
	Reverse	5′-TGA ATT CCA GAC TCC AGG CAC
**SREC-1**	Forward	5′-GAC TCC TTC TCA TCC GAT CC
	Reverse	5′-GGC GCG GAG GCT TAG GGA TGG
**CXCL16**	Forward	5′-CAT CAA TTC CTG AAC CCA TGG
	Reverse	5′-GAA TCG TCT CCG GAA ACA CCT
**MARCO**	Forward	5′-GGG AGA AAA AGG TGA AAG AGG
	Reverse	5′-TCC CCC AGG TAC CAC TGT AG
**SCARA3**	Forward	5′-TGC GGA TTC TTT ACC TCT TCC
	Reverse	5′-TTC GGA GAG AGA GTC CAC TTT T
**SCARA5**	Forward	5′-TGT GGG CAT CTT CAT CTT AGC
	Reverse	5′-CTC TCA TTC AGC CGG TTC AC
**COLEC12**	Forward	5′-CCA CGG TCA CCA TGA AAG A
	Reverse	5′-GCT TGT AAC CGA AGG ATT GC

### Silencing of SR by Small Interfering RNA (siRNA)

BEAS-2B cells were grown to 70% confluence in 24-well culture plates. SiRNA targeting CXCL16, LOX-1 and SR-B1 (Ambion, Applied Biosystems, Courtabeuf, France) or the negative control siRNA (25 nM) were mixed with Lipofectamine RNAiMAX solution (0.6 µl/well) (Life technologies, Saint Aubin, France). After 48 h, cells were activated with dsRNA for 24 h.

### Flow Cytometry

*A*nti-human LOX-1 and MARCO (Hycult biotechnology, Uden, The Netherlands), anti-human SR-A1 (Abnova Corp, Taipei, Taiwan), anti-human SR-B1 (BD Biosciences, San Diego, CA) mAb and goat polyclonal anti-human CXCL16 and anti-SREC-1 (R&D systems, Abingdon, UK) antibodies were used to analyze SR expression in 16 HBE cells and HBEC by flow cytometry. The specificity of the anti-SR antibodies was checked by using CHO cells transfected with the corresponding gene [31]. Endocytosis of FITC-conjugated acetylated-LDL (Ac-LDL) (Life Technologies), alexa-488-labelled mOVA or biotin-conjugated dsRNA was also evaluated. Results are expressed as median of fluorescence intensity (MFI) minus the value obtained with the relevant control (ΔMFI).

### Gene Reporter Assays

BEAS-2B cells were transfected with a NF-κB-luciferase and ISG-56-luciferase reporter plasmid (a generous gift of Dr Si Tahar, Tours, France) and stimulated with mOVA (50 µg/ml), dsRNA (10 µg/ml) or dsRNA/mOVA. Luciferase activity was measured in cell lysates using a luciferase activity kit (Roche Diagnostic). Results are expressed as counts per second.

### Protocols for dsRNA-induced Lung Inflammation

mOVA (100 µg), dsRNA (20 µg), dsRNA/mOVA, or PBS were administrated intra-nasally to C57BL/6 mice on day 0 (short exposure), or at days 0, 1 and 2 (repeated exposure). Inflammatory infiltrate was analyzed by total and differential counts and by histopathology study on lung sections. Phenotypic characterization was also performed by flow cytometry on lung and draining lymph nodes. Animals were handled and housed in accordance with the guidelines of the Pasteur institute Animal Care and Use Committee. All the experiments were performed after approval by the ethics committee for animal experimentation from the Nord–Pas de Calais Region (Agreement N°: 59-350163). Details are provided in File S1.

### Cytokine Quantification

The concentration of human CXCL8, CCL5 and CXCL10 (R&D systems), IFN-β (PBL, Piscataway, NJ) and of IL-29 (eBioscience, Paris, France) in BEC supernatants were determined by ELISA. Murine CCL5, CXCL10 and IL-12p70 concentrations were measured in bronchoalveolar lavages and protein lung extract by ELISA (R&D systems).

### Data Analysis

Statistical analysis was performed using the non-parametric Kruskal-Wallis test with the Dunn comparison (GraphPad San Diego, USA). Results with a p value less than 0.05 were considered as significant.

## Supporting Information

Figure S1 **MARCO, SR-A and SREC-1 surface expression in BEC and positive controls.** Unstimulated 16 HBE cells, HBEC and CHO cells transfected with the corresponding gene were analyzed by flow cytometry with the specific antibody (bold line). Isotype control is represented with dotted line. Histograms of one representative experiment out of 3 are presented.
(TIF)

Figure S2 **Binding of FITC-conjugated mOVA in HBEC.** Histograms of flow cytometry are reported. The filled line in upper and lower histogram showed the TNF-α- and PMA-stimulated cells (left and right histogram, respectively) as compared with cells in medium (bold line) and to the isotype control (dotted line). This is a representative experiment out of 3.
(TIF)

Figure S3 **Repeated administration of mOVA affects immune cell activation by poly(IC).** Mice received three intranasal injections of PBS, mOVA (100 µg), poly(I:C) (20 µg) or mOVA + poly(I:C). Lungs and lymph nodes were collected 24 h after the last injection. Data are expressed as the mean ± SEM from 3 independent experiments including 4 mice per group. (**A**) Numbers of CD86 positive macrophages, DC (total number and CD11c+) in the lung were shown. (**B**) Numbers of CD25 positive T lymphocytes (total number, CD4^+^ and CD8^+^ cells) in the lungs were reported. (**C**) Numbers of CD86 positive macrophages, DC (total number and CD11c+) in the draining lymph nodes were shown. (**D**) Numbers of CD25 positive T lymphocytes (numbers of CD4^+^ and CD8^+^ T cells) in the draining lymph nodes were reported.
(TIF)

File S1 (DOC)
