# Supplementary figures and images for: Scavenger Receptors in Human Airway Epithelial Cells: Role in Response to Double-Stranded RNA
Source: PLoS One. 2012 Aug 7;7(8):e41952. doi: 10.1371/journal.pone.0041952 (PMC3413698; doi:10.1371/journal.pone.0041952)

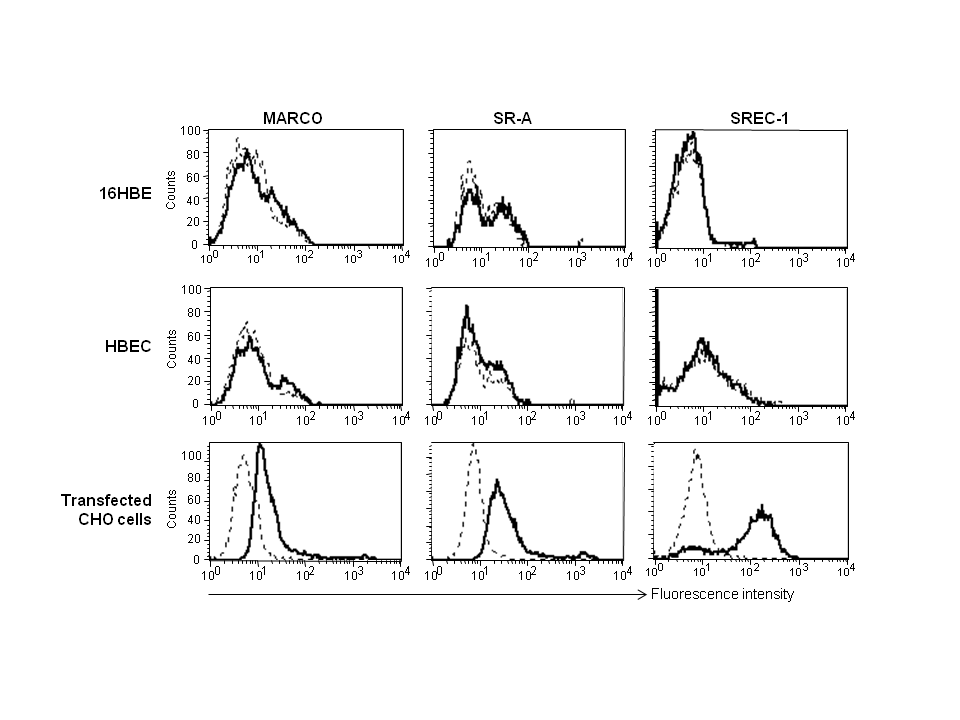

Supplement: Figure S1 — MARCO, SR-A and SREC-1 surface expression in BEC and positive controls. Unstimulated 16 HBE cells, HBEC and CHO cells transfected with the corresponding gene were analyzed by flow cytometry with the specific antibody (bold line). Isotype control is represented with dotted line. Histograms of one representative experiment out of 3 are presented. (TIF) [file pone.0041952.s001.tif]

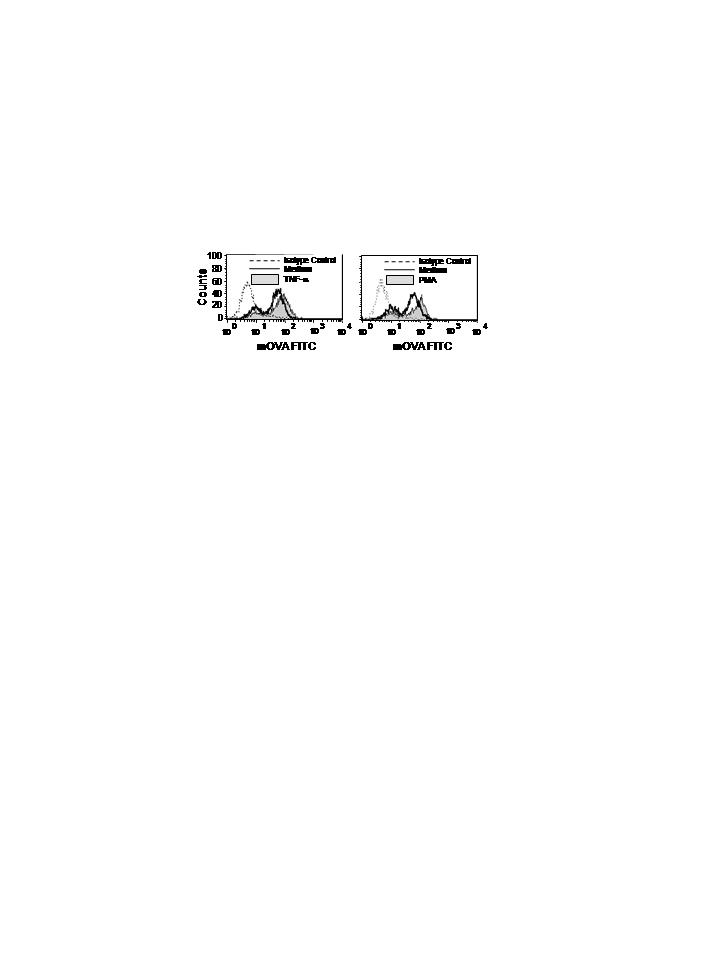

Supplement: Figure S2 — Binding of FITC-conjugated mOVA in HBEC. Histograms of flow cytometry are reported. The filled line in upper and lower histogram showed the TNF-α- and PMA-stimulated cells (left and right histogram, respectively) as compared with cells in medium (bold line) and to the isotype control (dotted line). This is a representative experiment out of 3. (TIF) [file pone.0041952.s002.tif]
