# Supplementary material for: Scavenger Receptors in Human Airway Epithelial Cells: Role in Response to Double-Stranded RNA
Source: PLoS One. 2012 Aug 7;7(8):e41952. doi: 10.1371/journal.pone.0041952 (PMC3413698; doi:10.1371/journal.pone.0041952)
Supplement: File S1 — (DOC) [file pone.0041952.s004.doc]

**Supporting information**

**Scavenger receptors in airway epithelial cells: role in response to double-stranded RNA**

Audrey Dieudonné, 1-6 David Torres, 1-5 Simon Blanchard, 7-.10 Solenne Taront, 2,3,11 Pascale Jeannin, 7-10 Yves Delneste, 7-10 Muriel Pichavant, 1-5 François Trottein,1-5 and Philippe Gosset 1-5

1Institut Pasteur de Lille, Center for Infection and Immunity of Lille, F-59019 Lille, France;

2Université Lille Nord de France, F-59000 Lille, France;

3CNRS, UMR 8204, F-59021 Lille, France;

4Institut National de la Santé et de la Recherche Médicale, U1019, F-59019 Lille,  France;

5Institut Fédératif de Recherche 142, F-59019 Lille, France;

­6 Service d’Hématologie-Immunologie-Cytogénétique, CH de Valenciennes, France;

7 LUNAM Université, Université d’Angers, Angers, France;

8 Inserm, Unit 892, Centre de Recherche en Cancérologie Nantes-Angers, Nantes, France;

9 CNRS, Unit 6299, Angers, France

10 Université d’Angers, CHU Angers, Laboratoire d’Immunologie et d’Allergologie, Angers, France.

11 Genomic and metabolic diseases, CNRS UMR8199, IBL, Lille, 59019, France ;

**Supporting Materials and methods**

*Reagents*

The following products were purchased from the indicated sources: fetal calf serum (FCS, Life technologies), Ultroser G (Life Science), agarose (Sigma-Aldrich, L’isle d’Abeau, France), Trizol reagent (Life Technologies), gelstar (Cambrex, Verviers, Belgium), and chloroquine (Sigma). PCR Primers (Table 1) were obtained from Eurofins MWG (Ebersberg, Germany). The different stimuli were obtained from the indicated sources: poly(I:C), Pam3CSK4, LPS, CpG, (Invivogen, San Diego, CA), IL-4, TNF-α and IFN-γ (R&D Systems, Abingdon, UK), PMA, ovalbumin and fucoidin (Sigma), AlexaFluor®488-labelled acetylated LDL (Life Technologies). To generate maleylated proteins, ovalbumin was successively detoxified, deglycosylated and then maleylated, as previously described [1]. Maleylated ovalbumin (mOVA) preparation contains no detectable endotoxin as detected by limulus anebocyte test and monocyte derived dendritic cell activation. mOVA was labelled with alexa-488 (Life technologies) for the endocytosis study. dsRNA was biotinylated with the Photoprobe® Biotin reagents from Vector laboratories (Peterborough, UK) according to their protocol. The following anti-human antibodies were used: mouse monoclonal antibodies (mAb) anti-human LOX-1, anti-human MARCO (Hycult biotechnology, Uden, The Netherlands), anti-human SR-A1 (Abnova Corp, Taipei, Taiwan), anti-human SR-B1 (BD Biosciences, San Diego, CA) and a polyclonal anti-human CXCL16 (R&D systems, Abingdon, UK). The following anti-mouse mAb were used to analyze cell recruitment in mice : FITC-labelled anti-mouse CD4, -CD11c, -120G8, -CD69, PE-conjugated anti-mouse CD8a, CD86, and APC-conjugated anti- CD3, anti-CD25, -I-Ad, PE-Cy5.5 anti-CD11b and -CD8a mAb (all from BD Biosciences, except 120G8 from Abcys, Paris, France). CXCL16-, LOX-1, SR-B1, SREC-1-specific and the negative control siRNA were purchased from Ambion (Woodward, Austin, TX). RNAiMAX lipofectamine and Fugene-6 was obtained from Life Technologies and Roche Diagnostic (Meylan, France), respectively. For the preparation of lung tissue, we used collagenase III and DNAse I (Roche Diagnostic, Rosny sous Bois, France), Immunohistofix and Immunohistowax (Aphase, Liège, Belgium).

*Primary culture of bronchial epithelial cells*

Human bronchial epithelial cells (HBEC) were prepared from bronchial wall explants dissected in healthy area of lung tissues obtained by resection in patients with lung cancer (n=6). One explant was placed on sterile plastic dishes coated with collagen G. After an adherence step, explants were cultured in DMEM-F12 supplemented with L-glutamine, 2% Ultroser G, and 2.5 µg/ml fungizon (Bristol laboratories) until confluence. Then, explants were transferred three times to new dishes to initiate new HBEC primary cultures. HBEC were transferred to collagen coated dishes in complete airway epithelial cell growth medium until confluent cells were obtained. Cell preparations are positive for cytokeratin staining and are not contaminated by fibroblast, macrophages and lymphocyte as determined by flow cytometry analysis.

After activation of BEC, cell supernatants were collected after 24 h. In some experiments, mRNA were collected 3, 6 or 24h after activation.

*RNA isolation and Reverse Transcriptase-Polymerase Chain Reaction (RT-PCR) analysis*

Total RNA was isolated from BEC after lysis in Trizol reagent (Life technologies) according to manufacturer’s instructions. RNA concentration was determined by spectrophotometry and its quality was evaluated by agarose gel electrophoresis with Gelstar staining.

Quantitative RT-PCR were performed using Superscript™ Platinum® SYBR® Green Two-step qRT-PCR Kit (Life technologies) according to manufacturer’s instructions. Real-time PCR was performed in an ABI-PRISM 7000 Sequence Detection System (Life technologies) to quantify the housekeeping gene β-actin, LOX-1, SR-A1, SR-B1, SREC-1 and CXCL16 mRNA. Cycle parameters were 50°C and 95°C for 2 min, followed by 45 cycles of 95°C for 15 s and 60°C for 30 s. The relative gene expression was calculated for each experiment after normalization using β-actin in folds (2-ΔΔCt) compared to unstimulated cells used as calibrator. Results were expressed as mean ± SEM.

*Flow cytometry*

*SR expression-* After activation,16HBE cells and HBEC were detached by treatment with 2 mM EDTA. After centrifugation, cells were resuspended in PBS with 2% FCS and were labelled (30 min, 4°C) with anti-LOX-1, MARCO, -SR-A1 and -SR-B1 mAb, goat anti-SREC-1 and -CXCL16 antibodies or the relevant control Ab. Bound antibodies were detected after incubation with phycoerythrin-conjugated rabbit IgG anti-mouse or anti-goat IgG (Southern Biotechnology, Birmingham, AL).

*Ligands endocytosis-* 16HBE cells were activated with 20 ng/ml TNF-α or10 nM PMA for 24h. Cells were then incubated for 30 min or 1h with FITC-conjugated acetylated-LDL (Ac-LDL), alexa-488-labelled mOVA or biotin-conjugated dsRNA (10 µg/ml). Cells were removed from culture plates by treatment with PBS plus 2 mM EDTA, centrifuged and resuspended in PBS with 2% FCS. Endocytosed dsRNA was detected, after cell permeabilization, using PE-labelled streptavidin (Life technologies). Inhibition experiments with unlabelled ligands were performed with a concentration of 50 µg/ml.

Flow cytometry analysis was performed on a FACSCalibur and data were analyzed using Cellquest software (BD Biosciences). Results were expressed median fluorescence intensity (MFI) after subtraction of the values obtained with the isotype control (ΔMFI).

*Gene reporter assays*

BEAS-2B cells were seeded at 8x10^4^ cells in 24-well plates 24 h before the experiment. Cell transfection was performed according to the manufacturer’s instructions using Fugene 6 transfection reagent (Roche) and 1 µg/well of a NFκB-luciferase reporter plasmid or of an ISG-56-luciferase reporter plasmid (a generous gift of Dr Si Tahar, Paris, France). After 24h, cells were cultivated in medium alone or stimulated for 24 h at 37°C with 20 ng/ml TNF-α or 1 ng/ml IFN-α2 (as a positive control) as well as with mOVA, dsRNA, or dsRNA/mOVA at the indicated concentrations.

*Protocols for dsRNA-induced lung inflammation*

Male C57BL/6 mice (8- to 12- wk-old) (Charles River laboratory, St-Germain sur l’Arbresle, France) were bred in specific pathogen-free animal facility. Mice were anesthetized and mOVA (100 µg per mouse), dsRNA (20 µg per mouse), dsRNA/mOVA, or PBS were administrated intranasally on day 0 (short exposure), or at days 0, 1 and 2 (repeated exposure). Mice were sacrificed 24 h after the last injection.

*Bronchoalveolar lavage (BAL)-* After mice euthanasia, lungs were canulated through the trachea and were washed twice with 500 µL PBS. Cellular fractions were processed for total and differential cell count. BAL fluids were assayed for cytokine and chemokine content.

*Lung tissue-* After collection of BAL fluids, lungs were perfused with PBS 2% FCS, and the left lobe of the lung was digested with collagenase III (1g/L) and DNAse I (2 UI/mL) (Roche Diagnostics) for 30 min at 37°C. Cell suspensions were then homogenized, centrifuged and resuspended in PBS/2% FCS for flow cytometry analysis. One right lobe was crushed with 1 mL PBS, 0.1% NP40 plus a cocktail of anti-proteases (Roche diagnostics) during 10 min at 4°C for protein and cytokine assays.

*Histopathology-* Moreover, a right lobe was fixed with Immunohistofix, included in Immunohistowax and was used for histological analysis. Lung sections were prepared and rehydrated. Granulocyte infiltration was analyzed by hematoxylin-eosin staining.

*Lymph nodes-* Mediastinal lymph nodes were collected, crushed, filtrated and resuspended in PBS/2% FCS. Total cell counts were determined.

*Phenotypic characterization of cells-* Cells were labelled (30 min at 4°C) with FITC-labeled anti-CD4, -CD11c, -120G8, -CD69 mAbs, PE-conjugated anti-CD8a, -CD86, -CD3, and APC-conjugated anti-CD3, -CD25 and -I-Ad mAbs, PE-Cy5.5 anti-CD11b and –CD8a mAb or isotype controls. mDC and pDC were respectively identified as CD11c+ I-Ad+ and 120G8+ I-Ad+ cells after exclusion of cells with high level of autofluorescence and then subdivided as CD11b+ or - cells, as previously reported [2]. Cells were washed and fixed with PBS containing 1% paraformaldehyde. Flow cytometry analysis was performed on a FACSCalibur (BD Biosciences) using Cellquest software.

**References**

1. Jeannin P, Renno T, Goetsch L, Miconnet I, Aubry JP, et al. (2000) OmpA targets dendritic cells, induces their maturation and delivers antigen into the MHC class I presentation pathway. Nat Immunol 1: 502-509.

2. Torres D, Dieudonne A, Ryffel B, Vilain E, Si-Tahar M, et al. (2010) Double-stranded RNA exacerbates pulmonary allergic reaction through TLR3: implication of airway epithelium and dendritic cells. J Immunol 185: 451-459.
